# Predicting the treatment outcomes of major depressive disorder interventions with baseline resting-state functional connectivity: a meta-analysis

**DOI:** 10.1186/s12888-025-06728-0

**Published:** 2025-04-07

**Authors:** Yanyao Zhou, Na Dong, Letian Lei, Dorita H. F. Chang, Charlene L. M. Lam

**Affiliations:** 1 Laboratory of Clinical Psychology and Affective Neuroscience, The University of Hong Kong, Hong Kong, China; 2 The State Key Laboratory of Brain and Cognitive Sciences, The University of Hong Kong, Hong Kong, China; 3 Brain and Behavior Laboratory, The University of Hong Kong, Hong Kong, China; 4 Department of Psychology, The University of Hong Kong, Hong Kong, China

**Keywords:** Major depressive disorder, Resting-state functional connectivity, Prediction

## Abstract

**Background:**

Current interventions for major depressive disorder (MDD) demonstrate limited and heterogeneous efficacy, highlighting the need for improving the precision of treatment. Although findings have been mixed, resting-state functional connectivity (rsFC) at baseline shows promise as a predictive biomarker. This meta-analysis evaluates the evidence for baseline rsFC as a predictor of treatment outcomes of MDD interventions.

**Method:**

We included MDD literature published between 2012 and 2024 that used antidepressants, non-invasive brain stimulation, and cognitive behavioral therapy. Pearson correlations or their equivalents were analyzed between baseline rsFC and treatment outcome. Nodes were categorized according to the type of brain networks they belong to, and pooled coefficients were generated for rsFC connections reported by more than three studies.

**Result:**

Among the 16 included studies and 892 MDD patients, data from nine studies were used to generate pooled coefficients for the rsFC connection between the frontoparietal network (FPN) and default mode network (DMN), and within the DMN (six studies each, with three overlapping studies, involving 534 and 300 patients, respectively). The rsFC between the DMN and FPN had a pooled predictability of -0.060 (*p* = 0.171, fixed effect model), and the rsFC within the DMN had a pooled predictability of 0.207 (*p* < 0.001, fixed effect model). The rsFC between the DMN and FPN and the rsFC within the DMN had a larger effect in predicting the outcome of non-invasive brain stimulation (-0.215, *p* < 0.001, fixed effect model) and antidepressants (0.315, *p* < 0.001, fixed effect model), respectively. Heterogeneity was observed in both types of rsFC, study design, sample characteristics and data analysis pipeline.

**Conclusion:**

Baseline rsFC within the DMN and between the DMN and FPN demonstrated a small but differential predictive effect on the outcome of antidepressants and non-invasive brain stimulation, respectively. The small predictability of rsFC suggested that rsFC between the FPN and DMN and the rsFC within the DMN might not be a good biomarker for predicting treatment outcome. Future research should focus on exploring treatment-specific predictions of baseline rsFC and its predictive utility for other types of MDD interventions.

**Trial registration:**

The review was pre-registered at PROSPERO CRD42022370235 (33).

**Supplementary Information:**

The online version contains supplementary material available at 10.1186/s12888-025-06728-0.

## Introduction

Major depressive disorder (MDD) is a prevalent psychiatric disorder that affects millions of patients worldwide [1]. The first-line intervention of MDD is antidepressants [2]. Many commonly used antidepressants, such as selective serotonin reuptake inhibitors (SSRIs) and selective serotonin and norepinephrine reuptake inhibitors (SNRIs), are based on the monoamine hypothesis, which proposes that depressive states are associated with a decrease in the concentration of monoamines (e.g., serotonin, norepinephrine, dopamine) in synaptic gaps [3, 4]. Therefore, effective antidepressants include those that alter the activity of post-synaptic serotonin receptors, such as serotonin modulators, as well as those that limit the reuptake of monoamine into the presynaptic cells, such as SSRIs and SNRIs [5]. Furthermore, antidepressants that target monoamine transmitters and try to increase the concentration of monoamine neurotransmitters in the synaptic gap, such as monoamine oxidase inhibitors [6, 7], are also effective in improving depression. Besides antidepressants that function according to the monoamine hypothesis, atypical antidepressants (e.g., bupropion, nefazodone) that alter the concentrations of more than neurotransmitters are also used as the treatment of depression [8].

Despite the popularity and importance of antidepressants, the efficacy of antidepressants remains limited because around 10% to 30% of depressive patients remain unresponsive to antidepressants [9]. There are alternative treatment options available for patients with MDD. For instance, cognitive behavioral therapy [10], and non-invasive brain stimulations such as repetitive transcranial magnetic stimulation [11], transcranial direct current stimulation [12], and electroconvulsive therapy [13]. Nevertheless, the effectiveness of non-pharmacological treatments of MDD, such as repetitive transcranial magnetic stimulation (rTMS), also remains heterogeneous [14].

Because depression is a complex and heterogeneous disorder [15–17], such a heterogeneous nature may be a probable reason for the limited treatment efficacy. Identifying patients who are likely to benefit from treatment and tailoring treatment selection to individual needs thus become essential for precision psychiatry. One way of boosting the precision of treatment is through biomarkers – functional variants or quantitative indexes with a biological basis that reflects the evolution of disease or predisposition to a disease or predicts treatment response [18]. Specifically, resting-state functional connectivity (rsFC) is a potential biomarker that is commonly studied.

One of the reasons why rsFC is a popular target of biomarker research is because it is independent from tasks and is more reproducible across scanning sites [19]. Moreover, rsFC has a solid neurobiological basis as it reflects structural connectivity [20]. Furthermore, many research of MDD have found alterations in rsFC [21–27], suggesting MDD as a disorder resulting from large-scale network dysfunction [28].

Currently, rsFC has been tested to differentiate patients from controls [29, 30], and predict remission in MDD [31, 32]. It has also been used to predict treatment response to electroconvulsive therapy [33] and psychotherapy [34] in depression. Nevertheless, neither the accuracy of disease state classification and prediction exceeds 90% and typically lies between 60%−70% [30, 32, 35].

Studies using connectome data to differentiate patients from controls suggested that not all inter- and intra-network rsFC connections contribute to the prediction equally, as certain rsFC connections, such as the rsFC within the emotion regulation circuit, play more important roles in prediction than others [36]. Therefore, before testing rsFC’s potential as robust biomarkers, it is important to figure out the most important type of inter- and intra-network rsFC connections based on specific prediction goal. Unlike previous studies that try to differentiate patients from controls, this review aimed to identify baseline rsFC characteristics that are consistently involved in the prediction of treatment outcomes.

Since we focused on the prediction of treatment outcome within patients only, which is measured continuously, rather than an effect size for measuring the differences between group means such as Cohen’s *d*, we chose Pearson *r*, a measure of the simple correlation between predictors and outcome [37]. Pearson *r* indicates the quantitative relationship between rsFC and treatment outcomes, thereby allowing us to rank different types of rsFC’s predictability on the treatment outcome based on their predictive strength. This quantitative approach is different from coordinate-based meta-analysis (e.g., [30]), which generates significant regions by spatially converging coordinates reported in different studies. We computed the pooled correlation coefficient between rsFC and treatment outcome. Specifically, we focused on the rsFC at baseline before any interventions were applied. This quantitative approach can identify which nodes and networks are most predictive of treatment outcomes. Based on our results, future machine learning studies could try assigning higher weights to nodes/networks that are more predictive of treatment outcomes to see whether their models’ predictability can be increased. Once we have a better understanding of the key rsFCs involved in predicting treatment outcome as well as their effect sizes, future studies could explore how demographic factors like age, gender, and disease trajectory might also influence treatment success, on top of the rsFC itself.

In sum, we reviewed integrated evidence from past studies which investigated the association between baseline inter- and intra-network rsFC patterns and symptom improvements following MDD interventions. We chose rsFC because it is a commonly examined indicator of the rs mode and could yield in higher number of final inclusions. Three types of MDD interventions are included in the current meta-analysis, namely, antidepressants, cognitive behavioral therapy (CBT), and non-invasive brain stimulation methods, including transcranial magnetic stimulation (TMS), transcranial direct current stimulation (tDCS), and electroconvulsive therapy (ECT). We chose these three types of interventions also due to their popularity in the field. We focused on the inter- and intra-network rsFC connections between or within eight networks as defined by the Human Brainnetome atlas [38]: subcortical network, visual network, somatosensory network, limbic network, dorsal attention network, ventral attention network, frontoparietal network, and default mode network. We calculated pooled correlation coefficients for inter- and intra-network rsFC connections that were reported in at least three separate articles/samples. With the synthesized evidence, this review investigated if the inter- and intra-network rsFC connection is consistently predictive on the outcome of MDD interventions. Furthermore, with the pooled Pearson *r*, this review summarized the overall strength of prediction that different patterns of rsFC have on the treatment outcome.

## Method

### Search & inclusion strategy

We targeted literature that examined the predictability of baseline rsFC on the treatment outcome of MDD interventions. We followed guidelines from the Preferred Reporting Items for Systematic Reviews and Meta-Analyses [39] in all aspects of design, conduct, and reporting. Two reviewers (Y.Z. and P.L.) independently performed the search in six key databases of psychology (i.e., PubMed, EMBASE, CINAHL, Web of Science Core Collection, Cochrane and ProQuest A&I) in December 2024. The review was pre-registered at PROSPERO CRD42022370235 [40].

This review covered all articles that were published between January 1st 2012 and December 10th 2024. This temporal span was chosen because the quality of neuroimaging technology has stabilized in later 2000s [41]. Our target studies were those that met the following criteria:

1. Recruited adult patients with MDD as the primary diagnosis. Other than anxiety symptoms or anxiety, patients with Axis I and Axis II disorders were excluded.
2. Acquired baseline rsFC for patients’ whole-brain during the resting-state by functional resonance imaging, did not derive rsFC using algorithms from external dataset. Used seed-based approaches such as ROI-to-ROI and seed-to-voxel, as well dimension reduction methods to derive rsFC;
3. Tested one of the target interventions of MDD (i.e., antidepressant, CBT, TMS, tDCS, ECT);
4. Tested the quantitative relationship between baseline rsFC and treatment outcome with measures of simple correlations such as Pearson *r* or its equivalents.

The complete search string was the linear combination of the search string of all individual parts: (depress* OR "major depressive disorder" OR MDD) AND.

("functional connectivity" OR connectivity) AND ((associat* OR predict* OR relat* OR modera* OR modulat*) AND (outcome OR response OR improv* OR respon* OR effect* OR efficacy)) AND (antidepressant OR SSRI OR SNRI OR "repetitive transcranial magnetic stimulation" OR rTMS OR tDCS OR "transcranial direct current stimulation" OR “ECT” OR “electroconvulsive therapy” OR "non-invasive brain stimulation" OR neuromodulation OR "cognitive behavioral therap*" OR "cognitive behavioural therap*" OR CBT). The search was conducted by screening the title and abstract of every paper in the target database.

Both reviewers independently included and excluded articles based on the inclusion and exclusion criteria. Articles were screened and included for further analysis if they met the inclusion and exclusion criteria mentioned above.

### Eligibility screening

After removing duplicates, two reviewers independently screened titles and abstracts of returned articles. Abstracts were included for further full-text screening if studies focused on adult patient sample, used one of the target interventions and measured rsFC by fMRI. Disagreements between reviewers were resolved through discussions based on the inclusion and exclusion criteria. A third arbitrator (C.L.) was consulted for impasses until a group consensus was reached.

After the abstract screening phase, both reviewers independently assessed the full text of each article to determine its eligibility based on our inclusion criteria. Both reviewers independently extracted information according to the inclusion and exclusion criteria from each article. A third reviewer (N.D.) independently verified the accuracy of the inclusion decision and extracted data. Articles were excluded if they failed to meet any of the inclusion criteria. Furthermore, articles were excluded if they were unavailable in full-text or were not written in English. Additionally, articles that studied late-life depression and psychotic depression (*N* = 2) as well as adopted a connectome or machine-learning classification approach (*N* = 6) were excluded. Studies reporting effect sizes or beta coefficients from multiple regression were excluded because the correlation between the strength of rsFC and treatment outcome is influenced by other variables. Articles contained non-Pearson measures of effect size such as beta coefficients were only included if these types of effect size could be converted to Pearson *r*. We checked reviews and meta-analyses for possible missingness in the inclusion.

### Data synthesis

Before conducting quantitative analysis and synthesis, the predictive coefficient of each type of network or node were extracted, if it was significant (*p* < 0.05). Similar to effect size indices such as Cohen’s *d*, which indicate the standardized mean difference, Pearson correlation coefficient is also a type of effect size and it measures the strength of association [42]. Predictive coefficients other than the Pearson* r* were converted to Pearson* r*, if the coefficients indicated one-to-one relationship (simple relationship) between the baseline rsFC and treatment outcome. Equations used in this review included $r\approx \rho =2 \times Sin(\rho \times \frac{\pi }{6})$ for converting Spearman’s ρ into Pearson *r* [43] and $r =\surd {R}^{2}$ for R^2^ reported in simple correlations. Beta coefficients in linear regression were only included and converted to Pearson correlation coefficients if they were from a simple linear regression where the standard deviations of the independent and dependent variable were available.

To generate a pooled correlation coefficient for each type of rsFC given the assorted nodes mentioned under each type of rsFC, we assigned extracted nodes to one of the eight functional networks based on the parcellation from the Human Brainnetome Atlas [38]. This atlas adds one additional network (i.e., the subcortical network) on top of the Yeo 7 Atlas [44]. The paper published by Zhang et al. [45] described the number of region of interest involved in each network in detail. The detailed information regarding how we categorized the nodes can be found in Supplement 1. In brief, the eight networks include 1) the subcortical network, which includes nodes such as the amygdala and hippocampus; 2) the visual network, which includes nodes such as the fusiform gyrus; 3) the somatosensory-motor network, which includes nodes such as the precentral gyrus; 4) the dorsal attention network, which includes nodes such as the inferior temporal gyrus; 5) the ventral attention/salience network, which includes nodes such as the insula; 6) the frontoparietal (FPN)/central executive network, which includes nodes such as the dorsolateral prefrontal cortex; 7) the limbic network, which includes nodes such as the orbital frontal gyrus; 8) the default mode network, which includes nodes such as the precuneus. For regions involved in multiple networks, we referred to previous literature of MDD, assigned the region to the network where the region is most frequently associated with, and ran sensitivity analysis to check the robustness of our result. For regions that were not explicitly mentioned in the atlas [38] or by Zhang et al. [45], we also referred to prior literature for categorization. For areas not mentioned in prior MDD literature, we referred to Yeo 7 atlas [44] for guidance in categorization.

Some articles presented multiple sets of correlations that could be categorized under the same type of rsFC connection (e.g., within-DMN rsFC). To avoid biasing the pooled Pearson *r* with multiple sets of correlation coefficients from the same study, we first averaged the correlation coefficients from the same article/sample that shared the same type of rsFC connection. If one study reported two coefficients that fell under the same type of rsFC connection, without averaging, the sample from that study would be used twice in the pooling process, as would the sampling variances. The predictive effects of that specific network on treatment outcome, as reported in that study, were also used twice. Therefore, averaging coefficients from the same study that fell under the same type of rsFC connection is necessary. When averaging multiple sets of coefficients from the same study that fell under the same rsFC connection, different coefficients did not receive different weights before averaging since they came from the same article and have the same sample size. Averaging was completed before pooling the coefficients of each type of rsFC connection across studies.

When pooling the results from multiple studies that examined the same type of rsFC, coefficients from different studies were weighted based on the sample size of the study. Coefficients from articles that contained a higher sample size would receive higher weights. For each type of rsFC connection (e.g., between DMN and FPN), the pooled Pearson *r* was generated if at least three articles correlated this type of rsFC at the baseline with the treatment outcome. We set this criterion because the result from at least two or more separate studies should be used in a meta-analysis [46]. Since we focused on three types of interventions, we wanted to avoid biasing the pooled result with the effect from a particular type of intervention. At the same time, we wanted to generate a relatively meaningful result given the small number of included studies. Therefore, instead of opting for a much more stringent criterion such as pooling from at least five articles, we generated the pooled Pearson *r* if the inter- and intra-network rsFC connection was reported in at least three studies.

Pooled Pearson *r* was generated for each type of the rsFC connection with the R package *‘meta’* in R [47]. The pooled correlation coefficient was generated for both the fixed and random effect models, which assume a constant or varying true effect of rsFC’s predictability on the treatment outcome across all included studies, respectively. This study did not have an a priori hypothesis regarding the nature of the true effect, and hence the results of both models were reported. After the generation of a pooled Pearson *r* for each type of rsFC connection, we compared the pooled correlation coefficients between different types of rsFC connections and different types of MDD interventions, if such statistics are available. I^2^ statistics were calculated to measure statistical heterogeneity across studies.

### Publication bias

Publication bias was examined with both the funnel plot and Egger’s regression intercept test. We included all studies from which the pooled Pearson correlation coefficients were generated. Data from studies reporting correlation coefficients for both types of pooled rsFC connections were averaged before generating the funnel plot and performing Egger’s regression test. During the assessment, Egger’s regression test examined the relationship between predictive strength and standard errors, with a significant result indicating a publication bias [48]. The funnel plot visualizes the level of symmetry by plotting standard errors of the coefficient reported in each study against the predictive strength [49].

## Results

### Literature search

**Fig. 1:**
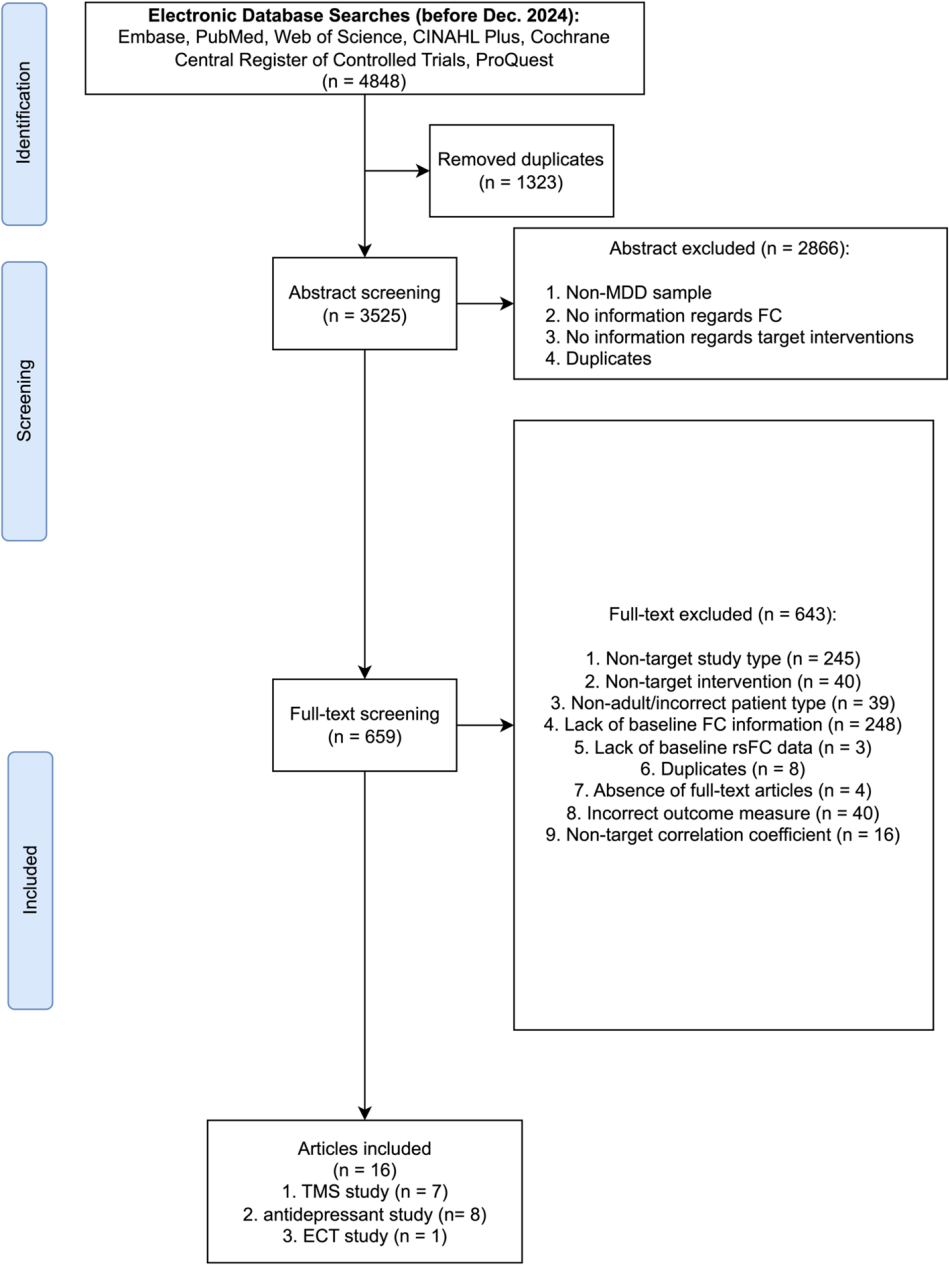
Flowchart of the study selection process

As shown in Fig. 1, six electronic databases returned 4848 records, of which 1323 were duplicates. During abstract screening, 2866 articles were excluded, resulting in 659 articles for the full-text screening. The final inclusions included 16 articles.

### Study characteristics

Pearson correlation coefficient or its equivalent was available in 16 studies, among which seven studies used TMS to treat MDD [50–56], eight used antidepressants to treat MDD [2, 45, 57–62], and one used ECT to treat MDD [33]. None of the included studies used CBT to treat MDD. Some included studies tested multiple MDD interventions.

As shown in Table 1, overall, most included studies, except for Elbau et al. [52] and Wang et al. [62], had a small sample size with fewer than 70 patients. Ten studies included in this review used treatment as monotherapy instead of add-on, meaning that the MDD treatment was the only treatment that the patient received at the time of intervention [2, 45, 53, 54, 57–62]. Five studies used treatment as add-ons, one of them is ECT study [33] and four of them are TMS studies [50, 52, 55, 56], meaning that ECT or TMS was added on top of current medications. One study [51] used rTMS as either monotherapy or add-on because the sample they recruited had a mixed status of current medication. TMS studies typically delivered the stimulation at 10 Hz for more than 1500 pulses in each session, though some studies used a higher intensity by using 3000 pulses per session. The number of sessions of TMS were within 10 to 25 sessions. The only included ECT study used ultra-brief right unilateral ECT and conservatively induced the stimulation at a dosage of either six times of the seizure threshold or maxim output, whichever was lower.

**Table 1:** Characteristics of included studies

**Authors**	**N (M/F)**	**Type of Depression**	**Severity of Depression**	**Age (Mean±SD)**	**Treatment Type (Target**^**l**^**)**	**Treatment Parameter**	**Type of rsFC Involved in Prediction**^**d**^	**Outcome Measure**	**Type of FC**	**Key Findings**
Avissar et al. (2017) [50]	9/18	Treatment-resistant (24 MDD, 3 bipolar II)	NA±NA^m^	42.1±NA	TMS (left DLPFC)	Add-on; 10 Hz, 3000 pulses/session * 25 sessions	FPN-VAN (*r* = 0.58)	HRSD-24	Seed-based FC	TMS successfully reduced depressive symptoms. Only baseline rsFC between the left DLPFC and the striatum predicted treatment efficacy.
**Cash et al. (2019) **[51]	24/19	Treatment-resistant (with no change in medication in the four weeks prior to screening)	34.58±6.67 (MADRS)	42.65±11.58	rTMS (left or right DLPFC)	Monotherapy & Add-on; NA Hz or pulse/session * 15 sessions. Responders: Five sessions of titrated left-sided treatment from week four to five. Non-responders: Randomized to left, right or sequential BL treatment starting from week four. 2000 pulses (L) or 1200 pulses (R) or 900 pulses (BL) followed by left 10 Hz rTMS/session.	DMN (*r* = -0.255)^f^; SMN (*r* = -0.21)	MADRS	Seed-based FC	Lower pre-treatment BOLD power in the caudate, PFC and thalamus correlate with better responsivity to treatment. Treatment response is also associated with FC in the DMN and affective network. Support vector model trained with aberrant patterns found by FC, BOLD signal power and disease-related parameters identified treatment responders with higher than 85% accuracy.
**Cui et al. (2021) **[2]	11/25	Unmedicated	21.86±3.25 (HRSD-17)	27.5±5.88	Escitalopram	Monotherapy; 5mg/day → 10-20mg/day (changed within 7 days)* 12weeks	DMN (*r* = -0.33)	HRSD-17	Seed-based FC	The rsFC within DMN’s core subsystem was significantly reduced at baseline. The rsFC within this subsystem increased after escitalopram treatment and become more similar to that of the healthy controls. However, the rsFC between dorsal medial PFC and medial temporal subsystems in patients remained reduced and abnormal after the treatment.
**Elbau et al. (2023)**^e ^[52]	118/177	Non-responsive to at least one antidepressant/Intolerable to at least two separate trials of antidepressants (was on stable antidepressant regimen for at least 4 weeks before treatment)	23.50±NA (HRSD-17)	42.89±NA	rTMS/iTBS (left DLPFC)	Add-on; rTMS: 10Hz, 3000 pulses/session * 20 sessions; iTBS: triplet 50 Hz bursts, repeated at 5 Hz, 600 pulses/session * 20 sessions	DMN-FPN (*r* = -0.16)	QIDS-SR	Seed-based + weight-map FC	The rsFC between subgenual ACC and stimulation site correlated with treatment outcome weakly but robustly, when the stimulated cortex was identified with the electric field modelling.
Fu et al. (2021) [53]	9/18	Treatment-naïve	21.81±5.04 (HRSD-17)	40.0±13.8	rTMS (left DLPFC)	Monotherapy; Unclear Hz, 1500 pulses/session * 10 sessions	FPN-VAN (*r* = 0.655)^g^	HRSD-17	Seed-based FC	The rsFC between left DLPFC and bilateral insula, and the structural connectivity between left DLPFC and left insular cortex significantly correlated with treatment outcome.
**Ge et al. (2020)**^**e**^[54]	21/29	Treatment-resistant (non-responsive to at least one adequate or two inadequate antidepressant trials during the current episode)	21.90±NA (HRSD-17)	43.66±NA	iTBS/HF-left stimulation (left DLPFC)	Monotherapy; iTBS: 50&5 Hz, 20 trains at bursts of 3 pulses at 50 Hz, bursts repeated at 5 Hz for 600 pulses/session * 20-30 sessions; HF-left stimulation: 10 Hz, 3000 pulses/session * 20-30 sessions	DMN-FPN (*r* = -0.62); DMN (*r* = 0.49)	HRSD-17	Seed-based FC	The rsFC between the subgenual ACC and right DLPFC negatively associated with treatment outcome. The rsFC between rostral ACC and left lateral parietal cortex positively correlated with treatment outcome. Identification of responders with these rsFC connections achieved a classification accuracy greater than 75% at different time points.
Harel et al. (2024) [61]	20/42	Treatment-naïve and treatment-seeking	16.95±5.85 (HRSD-21)	37.2±12.5	Sertraline or Fluoxetine or Escitalopram	Monotherapy; Sertraline: up to 200mg/day * 8 weeks; Fluoxetine/Escitalopram: unknown dosage * 8 weeks	SCN (*r* = -0.33)	HRSD-21	Seed-based FC	The reduction in the inter-hemispheric connectivity of medial amygdala negatively correlated with the severity of depression. Such an inter-hemispheric FC also predicted treatment response to antidepressants with an accuracy of 65.4%.
Hsu et al. (2021) [57]	5/17	Treatment-naïve	24.5±2.9 (HRSD-17)	40.70±11.6	Sertraline	Monotherapy; 25mg/day * 1week + 50mg/day * 5 weeks	VAN-SCN (rho = 0.09)^h^; DAN-SCN (rho = 0.48)	HRSD-17	Seed-based FC	Sertraline treatment restored the FC of the medial temporal lobe, the FPN, the thalamus, and the salience network. Abnormalities in the FC between the FPN and the core of DMN, between the FPN and salience network, and within the FPN persisted after treatment. The thalamo-prefrontal connectivity moderately predicted the treatment effectiveness.
Martens et al. (2021) [58]	15/19	Drug-naïve and drug-free	23.15±NA (HRSD-17)	30.29±NA	Escitalopram	Monotherapy; 10mg/day * 6 weeks	FPN-SMN (*r* = -0.412); DMN-VAN (*r* = 0.371)^i^	HRSD-17; BDI	ICA FC	The rsFC of the right FPN—with the posterior DMN (BL precuneus), and with the SMN and somatosensory association cortex correlated with symptom improvement positively.
**Moreno-Ortega et al. (2019) **[33]	NA/NA^j^	Treatment-resistant	26.5±3.9 (HRSD-24)	52.0±12.0	ECT (right unilateral, frontal)	Add-on; Conservative criteria (≥15 seconds). Ultra-brief right unilateral ECT. Threshold determined using dose titration method. Dose was either 6x seizure threshold or maxim output.	FPN – DMN (*r* = 0.685); FPN – Visual (*r* = -0.661); DMN (*r* = -0.699);	HRSD-24	Seed-based FC	Baseline reductions in FC between DLPFC and visual regions, as well as reductions in FC within the visual regions significantly predict symptom improvement after ECT. Visual dysfunction in depression is present.
Raij et al. (2023) [55]	8/17	Treatment-seeking (did not use antidepressant 4 months prior to study)	38.6±9.3 (BDI)	54.8±9.9	High frequency-rTMS (DLPFC)	Add-on; 10 Hz, 3000 pulses/session * 20 sessions^a^ or 10/20 Hz, NA pulses/session * 20-32 sessions^b^	FPN (*r* = 0.44)	BDI	Seed-based FC	The FC of the core network involving regions related to emotion-regulation and MDD-related DLPFC increased the repeatability of functional targeting more than the FC of subgenual ACC. The TMS targets selected based on the core network were constant within individuals, but varied between individuals.
**Wang et al. (2024) **[62]	28/59	Treatment-naïve	20.72±NA (HRSD-17)	27.07±NA	Escitalopram	Monotherapy; 5mg/day → 10-20mg/day * 12 weeks^c^	DMN-FPN (*r* = 0.35)^k^; DMN (*r* = 0.44)^k^;	HRSD-17	Seed-based FC	After 12 weeks of escitalopram, the remitter group showed significantly higher rsFC between subgenual ACC, DLPFC and inferior parietal lobule than the non-remitter group. The rsFC between subgenual ACC and the FPN is an effective predictor of antidepressant response.
**Weigand et al. (2018) **[56]	8/17	Treatment-resistant	38.6±9.3 (BDI)	54.8±9.9	High frequency-rTMS (DLPFC)	Add-on; 10 Hz, 3000 pulses/session * 20 sessions^a^ or 10/20 Hz, NA pulses/session * 20-35 sessions^b^	DMN-FPN (*r* = -0.51)	BDI	Seed-based	At the Boston site, stimulation sites that were more anterolateral predicted better antidepressant efficacy. Moreover, rsFC between the subgenual ACC and stimulation site negatively correlated with antidepressant efficacy. At the Michigan site, the only predictor of response to active but not sham rTMS was subgenual connectivity.
**Ye et al. (2022) **[59]	25/41	Treatment-naïve, treatment-washed (underwent a drug washout phase for at least 4 weeks)	23.11±NA (HRSD-17)	27.68±NA	Escitalopram or Venlafaxine	Monotherapy; Escitalopram: 10-20mg/day * 4 weeks; Venlafaxine: 75-225mg/day * 4 weeks	DMN (*r* = 0.42)	HRSD-17	ICA	Remitters showed significantly higher FC within the right angular gyrus of the DMN. This FC positively correlated with the reductions in symptom severity.
**Zhang et al. (2021) **[60]	25/34	Experiencing current depressive episodes with duration longer than 1 month but shorter than 2 years	22.71±4.60 (HRSD-17)	32.37±8.96	Escitalopram or Sertraline or Fluoxetine	Monotherapy; Escitalopram: 10-20mg/day * 12 weeks; Sertraline: 100-200mg/day * 12 weeks; Fluoxetine: 20-60mg/day * 12 weeks	DMN-FPN (beta = 0.26)	HRSD-17	Seed-based FC & k-means clustering	Treatment normalized FCs between DistalACC and left DLPFC, and between LateralACC and right amygdala. Abnormality remained for the FC between LateralACC and left amygdala.
Zhang et al. (2023) [45]	14/27	First-episode, recurrent (drug-naïve or have not taken more than 7 days of antidepressant in the past 14 days)	21.63±3.18 (HRSD-17)	26.15±4.76	Escitalopram	Monotherapy; 5mg/day → 10-20mg/day * 12 weeks^c^	SCN-VAN (rho = -0.344)	HRSD-17	Seed-based FC	Reduced connectivity between SCN and VAN at baseline increased in responders after treatment, and became similar to that of the controls. Reduced rsFC within the SCN and DMN remained dysfunctional after the treatment.

Bolded articles were used in pooled analysis, because at least three studies reported correlation coefficients for that particular type of rsFC connection.*ACC* anterior cingulate cortex, *BDI* Beck Depression Inventory, *BL* bilateral, *BOLD* blood oxygenation level-dependent, *DLPFC* dorsolateral prefrontal cortex, *DMN* default mode network, *FC* functional connectivity, *FPN* frontoparietal network, *HRSD-17/24* Hamilton Rating Scale for Depression (17 items/24 items), *iTBS* intermittent theta burst stimulation, *L* left, *MADRS* Montgomery-Asberg Depression Rating Scale, *NA* not applicable, *QIDS-SR* Quick Inventory of Depressive Symptomatology - Self-Report, *(r)TMS* (repetitive) transcranial magnetic stimulation, *R* right, *SCN* subcortical network, *SMN* somatomotor network, *VAN* ventral attention network

^a^Parameter used at the Michigan site

^b^Parameters used at the Boston site

^c^The dose of medication was adjusted by psychiatrist on the basis of patient response following the recommended range of 10-20mg/day

^d^Only connections involving up to two networks are presented. Connections involving more than three networks are not presented

^e^Both came from the THREE-D study [62], but were from different sites

^f^Averaged between posterior cingulate cortex- and subgenual cingulate cortex-seeded FC

^g^Averaged between L-DLPFC and right/left ACC

^h^Averaged correlation coefficient of two pairs of connections involving different nodes from the same connection type

^i^Only correlation coefficients related to HRSD-17 were shown

^j^The total sample size is 18

^k^Averaged between subgenual anterior cingulate cortex and left/right DLPFC (DMN-FPN) as well as left/right inferior parietal lobule

^l^For non-invasive brain stimulation studies only

^m^Greater than 16 (HRSD-24)

All studies that treated MDD with antidepressant used common antidepressants such as escitalopram and sertraline as monotherapy instead of add-on therapy. Antidepressants were delivered for at least four weeks, and up to 12 weeks among included studies. A commonly used dosage was 10–20 mg for escitalopram. Studies started escitalopram with different initial dosage, ranging from 5 to 10 mg per day. For studies that used sertraline, the initial dosage ranged from 25 to 50 mg per day, and the maximum dosage was 200 mg. For studies that used venlafaxine, clinicians typically started with 75 mg per day. The maximum dosage of prescription was 225 mg per day for venlafaxine. For fluoxetine, it was typically prescribed between 20–60 mg per day.

We did not find any eligible studies using CBT or tDCS, because during the full-text screening phase, articles that used these two interventions did not report a simple correlation coefficient indicating the relationship between baseline rsFC and treatment outcome and were thus excluded for not meeting the inclusion/exclusion criteria of this study.

### Frequently reported nodes in each network

From the 16 included studies, data from nine studies [2, 33, 51, 52, 54, 56, 59, 60, 62] were used to compute the pooled correlation coefficient for two types of rsFC connections: those within the DMN and those between the DMN and the FPN. It was because these two types of rsFC connections contained correlation coefficients from at least three studies. Data from seven included studies [45, 50, 53, 55, 57, 58, 61] could not be used to generate the pooled correlation coefficient because the rsFC provided in these studies did not contain at least three different sets of coefficients, failing to meet the paper's minimum condition for generating a pooled correlation.

**Fig. 2:**
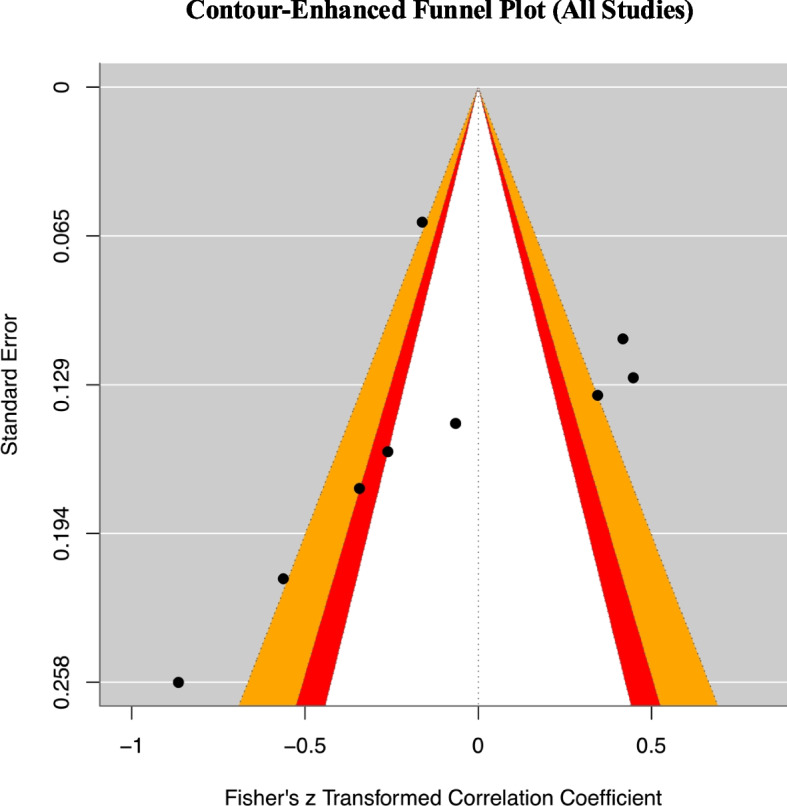
Contour-enhanced funnel plot. Contour-enhanced funnel plot containing data from studies that contained a coefficient indicating the relationship between treatment outcome and the strength of baseline rsFC within the DMN or between DMN and FPN

As shown in Fig. 2, we did not find evidence of a publication bias (*z* = −0.600, *p* = 0.549) when pooling across studies reporting both two types of rsFC. For studies reporting coefficients for both types of rsFC, we averaged the coefficients before performing the analyses of publication bias. For studies like that, we had performed the analyses with either of the coefficient for sensitivity check, and the conclusion remained the same.

**Fig. 3:**
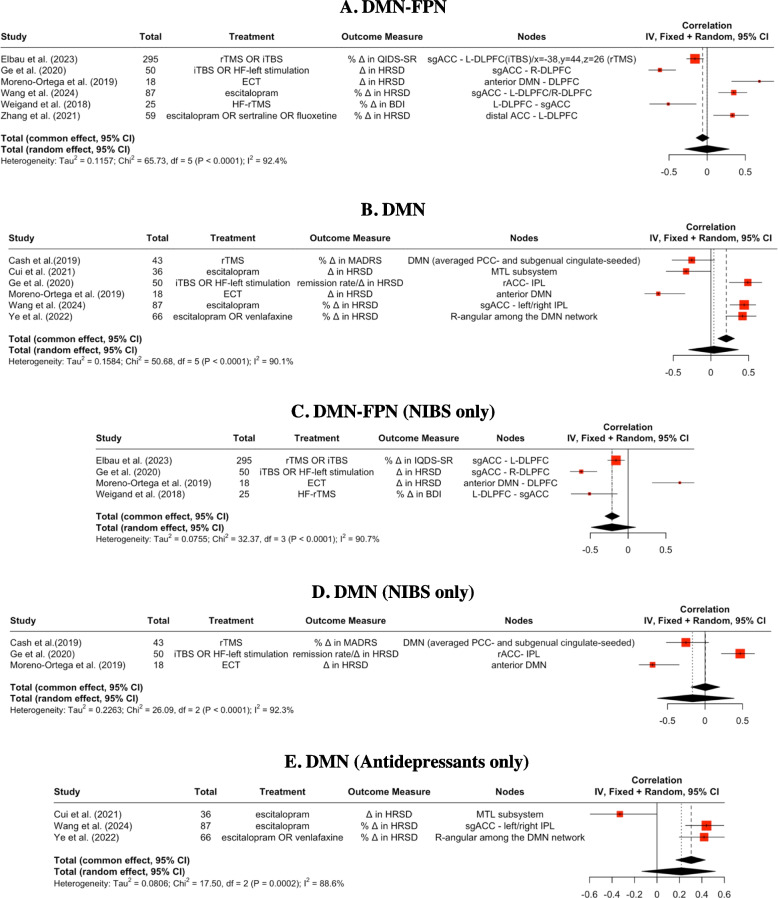
Pooled Pearson correlation coefficient for different types of rsFC connections. BDI = Beck's Depression Inventory; (DL)PFC = (dorsolateral) prefrontal cortex; DMN = default mode network; HF = high-frequency; HRSD = Hamilton Rating Scale for Depression; IPL = inferior parietal lobule; iTBS = intermittent theta-burst stimulation; MADRS = Montgomery-Asberg Depression Rating Scale; MTL = medial temporal lobe; PCC = posterior cingulate cortex; QIDS-SR = Quick Inventory of Depressive Symptomatology—Self-report; R/L = right/left; rTMS = repetitive transcranial magnetic stimulation; (sg)ACC = (subgenual) anterior cingulate cortex; △ = Changes

As shown in Fig. 3, the most frequently reported node in the FPN was dorsolateral prefrontal cortex (DLPFC). For DMN, the most frequently reported node was anterior cingulate cortex (often subgenual anterior cingulate cortex). For the details of nodes involved in the prediction of treatment outcomes and for details of node categorizations, please see Supplement 1.

### Contrasting directions of predictions from the rsFC of FPN-DMN and DMN

**Fig. 4:**
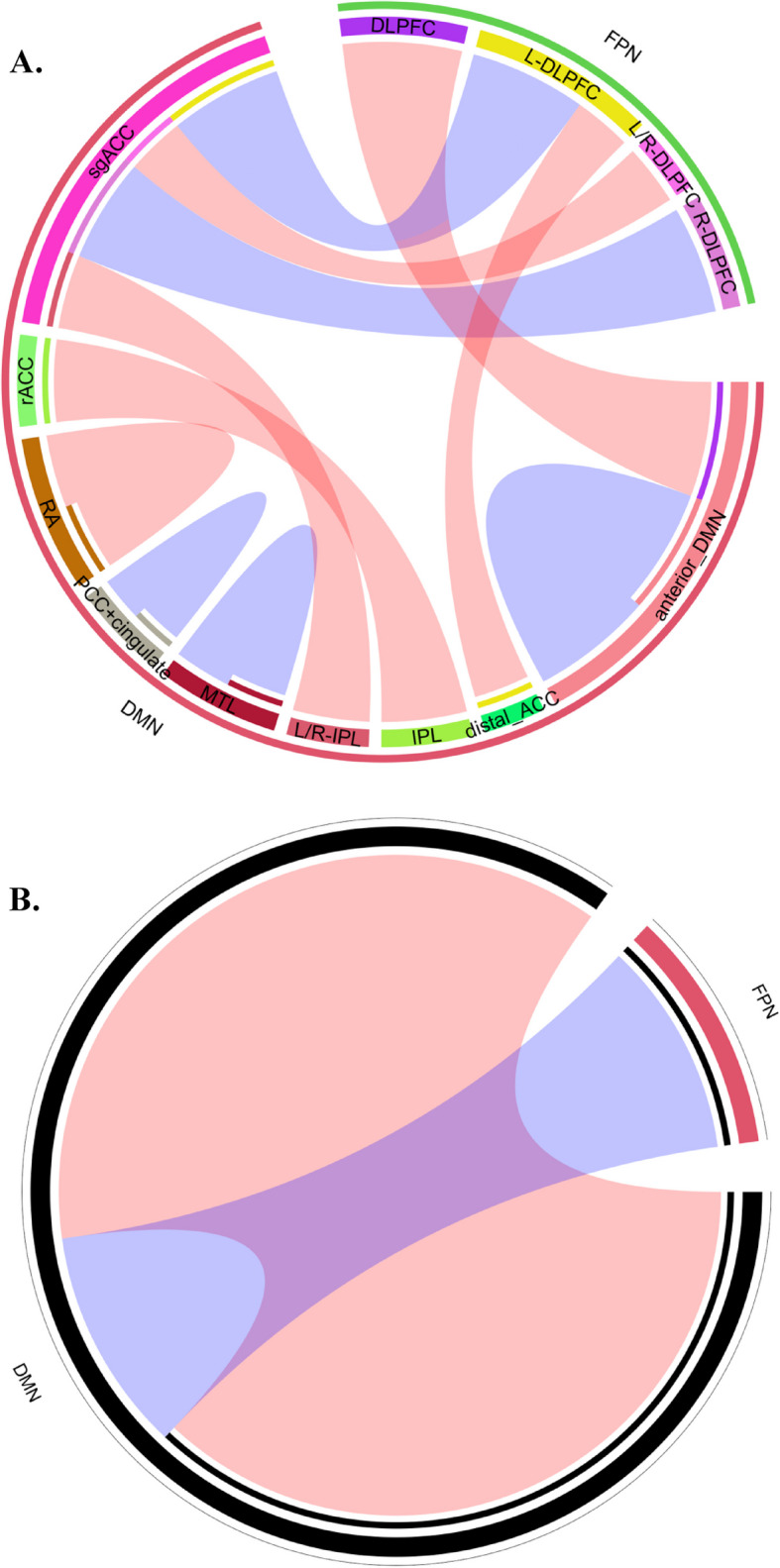
Circular layout of the rsFC connection. Visualization of (**A**) the predictability of individual nodes involved in each type of rsFC and (**B**) the predictability of each type of rsFC in general. Thickness of the lines is correlational to the strength of the connection. Blue lines represent negative connectivity between or within nodes, whereas red lines represent positive connectivity between or within nodes. DMN = default mode network; FPN = frontolarietal network; IPL = inferior parietal lobule; (L-/R-)DLPFC = (left/right) dorsolateral prefrontal cortex; MTL = medial temporal lobe; PCC = posterior cingulate cortex; RA = right angular part of the DMN; rACC = rostral anterior cingulate cortex; sgACC = subgenual anterior cingulate cortex;

Based on the 16 studies included, the pooled Pearson correlation coefficient was generated for the rsFC within the DMN and between the DMN and FPN from nine studies. Types of rsFC connection, strength of the pooled correlation, nodes involved, and their strength of prediction were shown in Figs. 3 and 4A. Other types of rsFC connections, such as the connection between FPN and ventral attention network, did not contain different sets of Pearson *r* from at least three different samples/studies for the generation of a pooled correlation coefficient. The predictive rsFC connections on the treatment outcome that were not used in the generation of the pooled correlation were presented in detail in Supplement 2.

Following the same principle for the generation of a pooled correlation coefficient, treatment-specific correlation coefficients were generated in addition to treatment-general pooled correlation coefficients, for eligible interventions. Specifically, for the rsFC within the DMN, treatment-specific pooled correlation coefficients were generated for predicting the outcome of non-invasive brain stimulations and antidepressants. For the rsFC between the DMN and FPN, treatment-specific pooled correlation coefficient was generated for predicting the outcome of non-invasive brain stimulations.

As shown in Fig. 4B, for the direction of prediction, the pooled rsFC between the FPN and DMN predicted treatment outcome negatively, meaning that patients with weaker baseline rsFC between the FPN and DMN tended to have higher symptom improvement after MDD interventions. On the contrary, the pooled rsFC within the DMN predicted the treatment outcome positively, meaning that patients with higher baseline rsFC within the DMN are more likely to have higher symptom improvement after interventions.

### Predictive strength of between-network connectivity

The pooled predictive strength was generated for the connectivity between the DMN and FPN. As shown in Table 2, for the predictive strength that baseline rsFC between the DMN and FPN has on the treatment outcome of MDD interventions, we found a pooled correlation coefficient of −0.060 (95% CI: −0.147 – 0.026, *p* = 0.171) for the fixed effect model, which assumes a constant true effect of rsFC’s predictability on the treatment outcome across all included studies. For the random effect model that assumes a varying treatment outcome from study to study, the pooled predictive strength was −0.003 (95% CI: −0.303 – 0.298, *p* = 0.987). Between-study variability was large, as indexed by a heterogeneity (I^2^) of 92.4%, indicating a large variability and inconsistency in the predictive power of rsFC between brain networks on the treatment outcome.

**Table 2:** Summary of Pooled Pearson Correlation for Each Type of Connection

Type of Connection	Number of Studies	Total Sample Size	Outcome Measure	Pooled Correlation (95% CI)		I^2^(%)	τ^2^
				Fixed Effect Model	Random Effect Model		
DMN-FPN	6	534	Changes in Depression Severity	−0.0603 (−0.1466; 0.0260) *p* = 0.1706	−0.0025 (−0.3025; 0.2975) *p* = 0.9869	92.4	0.1157
DMN	6	300	Changes in Depression Severity	0.2073 (0.0905; 0.3240) ***p* = 0.0005**	0.0386 (−0.3059; 0.3831) *p* = 0.8263	90.1	0.1584
DMN-FPN (NIBS only)	4	388	Changes in Depression Severity	−0.2154 (−0.3165; −0.1144) ***p* < 0.0001**	−0.2165 (−0.5331; 0.1001) *p* = 0.1802	90.7	0.0755
DMN (NIBS only)	3	111	Changes in Depression Severity	0.0046 (−0.1894; 0.1987) *p* = 0.9627	−0.1696 (−0.7493; 0.4101) *p* = 0.5664	92.3	0.2263
DMN (Antidepressants only)	3	189	Changes in Depression Severity	0.3148 (0.1687; 0.4609) ***p* < 0.0001**	0.2209 (−0.1358; 0.5775) *p* = 0.2248	88.6	0.0806

### Predictive strength of within-network connectivity

The pooled predictive strength of the rsFC within DMN was synthesized in this review among six studies using the rsFC of nodes in the DMN to predict the treatment outcome of MDD interventions. Overall, as shown in Table 2, we found a pooled predictive strength of 0.207 (95% CI: 0.091 – 0.324, *p* < 0.001) for the fixed effect model. A predictive strength of 0.039 (95% CI: −0.306 – 0.383, *p* = 0.826) was found for the random effect model. Large variability between studies was found for the predictive strength of within-network connectivity, with a heterogeneity (I^2^) of 90.1%, pinpointing a large variability and inconsistency in the predictive power of rsFC within DMN on the treatment outcome.

### Predictive strength of between-network connectivity by treatment type

For the predictive effect of the rsFC between the DMN and FPN on non-invasive brain stimulations (i.e., TMS, ECT), the pooled Pearson correlation yielded a predictive strength of −0.215 (95% CI: −0.317 – −0.114, *p* < 0.001) and −0.217 (95% CI: −0.533 – 0.100, *p* = 0.180) for the fixed effect and random effect model, respectively. The heterogeneity and variability across all included studies that predicted the effect of non-invasive brain stimulations with the rsFC between the DMN and FPN were large, with a heterogeneity (I^2^) of 90.7%. This was smaller than the I^2^ of studies that used the rsFC between the DMN and FPN to predict treatment outcomes, irrespective of the specific intervention being non-invasive brain stimulation, which was 92.4%.

For the predictive effect of the rsFC within the DMN on non-invasive brain stimulation, we found a pooled correlation coefficient of 0.005 (95% CI: −0.189 – 0.199, *p* = 0.963) and −0.170 (95% CI: −0.749 – 0.410, *p* = 0.566) for the fixed effect and random effect models, respectively. Large variability was found among studies predicting the effect of non-invasive brain stimulation with DMN, with a heterogeneity (I^2^) of 92.3%, which was greater than that of the treatment-general prediction. For the predictive effect of the rsFC within the DMN on antidepressants, the fixed effect and random effect models yielded a predictive strength of 0.315 (95% CI: 0.169 – 0.461, *p* < 0.001) and 0.221 (95% CI: −0.136 – 0.578, *p* = 0.225), respectively. Large variability was found among studies with a heterogeneity (I^2^) of 88.6%, which was smaller than that of the treatment-general prediction.

### Connections from which a correlation coefficient could not be pooled

Data from seven studies were not used in the pooled analysis because many types of rsFC connections contained less than three studies to generate the pooled correlation coefficient. Two studies predicted the treatment outcome with rsFC between the FPN and ventral attention network (VAN), and found a large effect size [50, 53]. One study predicted the treatment outcome with rsFC between the DMN and VAN, and found a medium predictability of rsFC [58]. One study predicted the treatment outcome with rsFC within the FPN and reported a medium to large effect size [55]. Other types of connections involved networks such as the subcortical network and somatomotor network, and some connections even involved connections between more than two networks. For details of all the nodes and their corresponding predictability on treatment outcomes, as reported in all eligible articles, please see Supplement 2.

## Discussion

In this review, we examined whether inter- and intra-network rsFC at the baseline predicted the outcome of MDD treatments. Overall, this review included 16 studies that were published between January 2012 and December 2024, collectively reporting on 892 MDD patients and two treatment modalities (i.e., antidepressant, non-invasive brain stimulation). We further pooled result from 679 patients, nine studies, and two types of rsFC: within DMN as well as between DMN and FPN. Our findings suggested a small predictability of the baseline rsFC on the treatment outcome of MDD interventions. For the rsFC between the FPN and DMN, the pooled Pearson correlation coefficients were −0.060 (95% CI, −0.147 – 0.026, *p* = 0.171) and −0.003 (95% CI, −0.303 – 0.298, *p* = 0.987) for the fixed and random effect models, respectively. For the rsFC within the DMN, the pooled Pearson correlation coefficients were 0.207 (95% CI: 0.091 – 0.324, *p* < 0.001) and 0.039 (95% CI: −0.306 – 0.383, *p* = 0.826) for the fixed and random effect models, respectively. We found a higher predictive power of rsFC within the DMN on antidepressants (0.315, *p* < 0.001, fixed effect model) and between the DMN and FPN on non-invasive brain stimulations (−0.215, *p* < 0.001, fixed effect model).

Overall, as illustrated in Fig. 4B, different types of rsFC at baseline predicted the treatment outcome in different directions: The rsFC between the DMN and FPN predicted the treatment outcome negatively, whereas the rsFC within the DMN predicted the treatment outcome positively. Relative to treatment-general effects, stronger predictive effects were found in the rsFC between the DMN and FPN for non-invasive brain stimulations, and within the DMN for antidepressants. However, the stronger treatment-specific effect was still small in both the fixed and random effect models. In the remainder of this Discussion, we will discuss interpretations of the results, and compare our findings with previous research, focusing both on similarities and differences.

### The baseline rsFC's predictive effect was small

In general, we found a small predictive effect of baseline rsFC on treatment outcome. Despite all extracted coefficients were significant predictors of treatment outcome, as reported in the original article, the 95% confidence interval of the pooled correlation of rsFC between the DMN and FPN included 0. The pooled correlation of the rsFC between the DMN and FPN was thus insignificant, when it was used to predict general treatment outcome irrespective of treatment modality. The pooled correlation of the rsFC within the DMN was significant in making both treatment-general prediction and antidepressant-specific prediction. The rsFC between the DMN and FPN was significant in predicting the outcome of non-invasive brain stimulation. Our results thus partially in line with prior literature, which stated that rsFC is predictive of the treatment outcome of MDD interventions. Specifically, we found that different types of rsFC connections predicted treatment outcomes differently depending on the type of treatment they were predicting.

Despite the final inclusion only reporting two types of treatments (antidepressants and non-invasive brain stimulation), treatment protocols varied between studies. Even though all TMS studies used excitatory stimulation and primarily targeted DLPFC, mainly the left DLPFC, and all antidepressant studies used either SSRIs or SNRIs, the heterogeneities in treatment duration and intensity/dosage might still have an impact on the final pooled results. Subgroup analysis based on different treatment protocols was infeasible due to the small final inclusions.

We found stronger treatment-specific effects in the rsFC between the DMN and FPN for non-invasive brain stimulations. Furthermore, the pooled effect reported in the fixed effect model (−0.215) was significant. It was reported previously that the strength of rsFC between the DMN and FPN (subgenual anterior cingulate cortex – dorsolateral prefrontal cortex) correlates with the treatment outcome following TMS intervention [63]. Moreover, DMN was found to be predictive on the outcome after antidepressant treatment [64] and TMS [65]. These pieces of evidence are in line with our findings that pinpoint the importance that the rsFC of DMN and FPN has in predicting the treatment outcome.

Considering the importance of DMN-FPN connection in MDD [28], the small predictability of the rsFC between the DMN and FPN on the treatment outcome was unexpected. Nevertheless, the result was understandable because of the sample characteristics. For the rsFC connection between DMN and FPN, all patients recruited in antidepressant studies were treatment-naïve, whereas those recruited in the non-invasive brain stimulation studies were treatment-resistant. It was found that refractory and nonrefractory patients shared distinct abnormalities in rsFC [66, 67]. Even though some patients reached to remission after treatment, separate correlation coefficients were not reported by included studies, and hence subgroup analysis according to the refractory status was impossible. Since the course of the disease is associated with differences in neural activities [68, 69], treatment-naïve and treatment-resistant patients are likely to have different baseline rsFC, resulting in a small predictability of rsFC after pooling. Previous research has shown that resistant and non-resistant patients have different limbic activities. However, none of the included studies reported the predictability of nodes from the limbic network. As a result, the rsFC between the DMN and FPN may not be sufficient to capture all patients’ functional abnormalities.

Furthermore, antidepressants and non-invasive stimulation appear to have different mechanisms, as antidepressants alter neural activity in the amygdala [70, 71], add-on TMS alters the left middle temporal cortex and fusiform gyrus [72], and ECT response is associated with FC between the hippocampus and ventromedial prefrontal cortex [73]. Indeed, the treatment-specific pooled correlation of DMN-FPN was not only greater than that of the treatment-general correlation but also significant. Since the treatment-specific pooled correlation was generated based on non-invasive brain stimulation studies, focusing on the same type of treatment thus enhances the predictability of the rsFC.

The small predictability of the rsFC within the DMN was also unexpected given the importance of DMN in depression [28, 74]. One possible explanation is that distinct subregions of the DMN might show differential response to treatment. To substantiate, DMN was found to consist of several distinct subsystems, including the anterior DMN centered on the medial prefrontal cortex (PFC) and the posterior DMN centered on the posterior cingulate cortex and precuneus [24]. In treatment-resistant depression, the hyperconnectivity within the posterior DMN normalized after antidepressant treatment, whereas the abnormality in the anterior DMN persisted [75]. More negative FC between the anterior cingulate cortex and subcallosal cortex was found to correlate with better symptom improvement [76]. In one of our included studies, even subregions within the anterior DMN showed different correlations with treatment outcome [33]. It is thereby reasonable to observe a relatively weak predictive effect from the rsFC within the DMN on the treatment outcome when we combined the correlation coefficients from nodes belonging to both the anterior and posterior portions of the DMN. Considering the heterogeneity among different subregions within the DMN, the fact that we were able to obtain an overall small to moderate effect of prediction is still promising. Nevertheless, future research is needed to further examine if different subregions within the DMN indeed predict treatment outcomes differently.

Similar to the rsFC between the DMN and FPN, we also found the rsFC within the DMN to have a larger effect in predicting the outcome of antidepressants. This result was not surprising because antidepressants were found to normalize activity within the DMN in patients with chronic depression [77]. Furthermore, DMN’s activity during tasks was also found to predict the outcome of antidepressants [78]. Nevertheless, the rsFC within the DMN was small and insignificant in predicting the treatment outcome of non-invasive brain stimulations (0.005, *p* = 0.963, fixed effect model). The differential treatment-specific prediction that rsFC within the DMN had on antidepressants and non-invasive brain stimulation thus suggested the importance of running treatment-specific prediction with baseline rsFC in the future.

### Distinct roles of DMN and FPN in MDD may contribute to the contrasting direction of prediction

Contrasting directions of prediction were found among different types of rsFC. The rsFC between the DMN and FPN predicting the outcome of different modalities of MDD interventions negatively, whereas the rsFC within the DMN predicting the treatment outcome positively. The sign of prediction in the pooled Pearson correlation for the two rsFC connections (DMN-FPN, DMN) is consistent with our expectations and with the finding of Long and colleagues [79]. In their study, hyperconnectivity within the DMN at the baseline was associated with treatment improvement. In our study, the strength of the baseline rsFC within the DMN also positively correlated with the treatment outcome. The negative correlation between the treatment outcome and the strength of baseline rsFC between the FPN and DMN has previously been reported, albeit in mild to moderate depression [80]. Taken together, the opposite directions of predictions between the rsFC within the DMN and between the DMN and FPN probably imply that patients with poorer control of the DMN by the FPN [81] and a hyperactive DMN [82] at the baseline are more likely to benefit from MDD treatments.

### Small effect size does not equate to a limited predictability

In this review, we extracted coefficients that indicate the predictive power of rsFC on the treatment outcome of MDD interventions. The targeted treatment outcomes in this review were changes in depression severity. However, the limited effect size does not necessarily imply that rsFC is limited in predicting treatment outcomes. This is because depression severity, quantified by questionnaires, which was commonly used as the measure of treatment outcomes in the included studies, may not be the most accurate indicator treatment success.

MDD patients can be classified into subgroups with distinct symptom clusters, each exhibiting a unique rsFC profile [83]. Although these rsFC-based subtypes were found to correlate with variations in specific depressive symptoms such as anhedonia and insomnia, they did not correlate with differences in overall depression severity. Moreover, different types of interventions were found to be effective in improving specific depressive symptoms. For instance, rTMS was most effective in improving executive function [84], while escitalopram targeted core depressive symptoms such as anhedonia, and tDCS addressed sleep/insomnia problems [85]. There has already been some preliminary evidence suggesting that baseline rsFC between the nucleus accumbens and regions such as the supplementary motor area and anterior cingulate cortex could predict changes in anhedonia 16 weeks after the antidepressant treatments [86]. Correlating rsFC with particular symptoms, separated by the type of MDD interventions, might yield a different, probably even a higher predictability. Nevertheless, more study is needed to test this speculation, as the existing evidence regarding baseline rsFC’s predictability on the improvement of distinct symptoms of depression remained limited.

Since our review focused on the predictability of baseline rsFC on treatment outcomes, which were measured on a continuous scale (e.g., changes in Hamilton Rating Scale for Depression), the small effect size does not necessarily imply that rsFC is useless in predicting treatment outcomes that are measured categorically. A recent review examined if baseline whole-brain connectome data could predict treatment outcome, which was defined categorically with criteria such as ≥ 25% reduction in depression severity [87]. The baseline connectome data was found to have an overall classification accuracy of 77% for binarily defined treatment outcomes among patients with internalizing disorders, mostly depression.

Furthermore, the small predictability of the rsFC within the DMN and between the DMN and FPN did not imply that the predictability of other types of inter- and intra-network rsFC connections was also limited. To substantiate, the baseline rsFC between the FPN and VAN appeared to have a large effect (0.7) in predicting the treatment outcome. However, this finding was not derived from pooled analysis, as this type of rsFC connection contained two instead of three eligible studies for generating the pooled coefficient. Therefore, future research examining the predictive role of the rsFC between the FPN and VAN, as well as other inter- and intra-network connections, on treatment outcome is necessary.

Lastly, since baseline rsFC showed differential effects in making treatment-general and treatment-specific predictions, with the latter greater than the former in most cases, future studies should continue investigating the predictive role of baseline rsFC on treatment outcomes. It is thus too early for us to conclude the limited predictability of baseline rsFC on treatment outcome. Future research could also consider combine deep learning approaches with the technique of explainable artificial intelligence, as such a combination appeared to be effective in identify FC patterns that are most predictive of treatment outcome [88].

## Limitations

Five features among included studies might limit the conclusions that we could draw from this meta-analysis. First, the sample of included studies was small and varied. Most studies had samples less than 70, with the smallest sample being 18. The small sample size thereby contributes to a wide confidence interval of pooled correlation coefficients and weakens the overall effect of prediction. Future studies that study the same type of treatment are encouraged to collaborate with each other to increase the sample size of the investigation.

Second, the included studies in our study differ in sample characteristics, study designs, and imaging processing pipelines. These heterogeneities will thus limit the generalizability of our findings to the population with MDD. If more studies report the predictability of baseline rsFC on the treatment outcome, subgroup analyses could be performed to test for the influence of study designs, treatment modalities, and imaging processing pipelines on rsFC’s predictability. Future studies that have collected baseline rsFC in MDD patients are thus encouraged to report such indices. Furthermore, the predictability of the baseline rFC could be further studied if the imaging processing pipelines could be standardized and refined. Future studies are hence encouraged to follow standard imaging processing pipelines and finely delineate the differential predictability of distinct subregions of a network on treatment outcome.

Third, our inclusion/exclusion criteria were stringent; for instance, we excluded studies that used the whole-brain connectome data to predict treatment outcome. Although the stringent criteria were set to ensure our result reflected the predictive effect of baseline inter- and intra-network rsFC on the outcome of depression treatment, these criteria resulted in a relatively low number of final inclusions.

Fourth, although we targeted tDCS and CBT in our meta-analysis, none of the studies using tDCS and CBT met our inclusion criteria. Our findings are thus mainly driven by the prediction effect of rsFC on the effects of non-invasive brain stimulation, mainly TMS, and antidepressants. As a result, the conclusions drawn from our meta-analysis remain limited to these two types of interventions for MDD at the moment. In accordance with the second limitation, future studies of tDCS and CBT are encouraged to correlate the baseline rsFC with treatment outcomes. More studies in tDCS and CBT are necessary to comprehensively test for baseline rsFC’s predictability on treatment outcome. Because CBT is a widely utilized treatment for depression, this information is also highly relevant to the field, and has the potential to improve the precision of treatment.

Lastly, it is important to note that most studies focused on the predictive effects of only one or two nodes on treatment outcomes. In an attempt to offer a comprehensive and systematic summary of the predictive strengths of resting-state neural predictors on treatment outcomes, we adopted a network-based approach. This involved manually assigning nodes to designated networks to achieve a balance between the variety of reported nodes and the specificity of conclusions drawn from this study. Therefore, it remains unknown whether the remaining nodes within the network are equally predictive as those reported in this review.

## Conclusion

Overall, we found a small predictive effect that rsFC has on the treatment outcome of MDD interventions. Networks involved in prediction include the FPN and DMN. The most frequently reported nodes in prediction include DLPFC in the FPN and (subgenual) anterior cingulate in DMN. In general, MDD patients with higher rsFC within the DMN or lower rsFC between the FPN and DMN at the baseline improve more after the MDD intervention. The rsFC within the DMN had a small but significant effect in predicting the general treatment response for MDD interventions. While the rsFC within the DMN demonstrated slightly higher predictive power for antidepressant outcomes, the overall effect remained small. In contrast, the rsFC between the DMN and the FPN showed an insignificant effect in predicting general treatment responses for MDD. However, it exhibited a small but significant effect in predicting outcomes specifically for non-invasive brain stimulation therapies. Our pooled evidence thus suggested that baseline rsFC within the DMN and between the DMN and FPN had a small and differential predictability on the outcome of antidepressants and non-invasive brain stimulation, respectively.

## Supplementary Information

Supplementary Material 1.

Supplementary Material 2.

## Data Availability

Data is provided within the supplementary information files.

## Abbreviations

CBT: Cognitive Behavioral Therapy
CI: Confidence Interval
(DL) PFC: (Dorsolateral) Prefrontal Cortex
DMN: Default Mode Network
ECT: Electroconvulsive Therapy
fMRI: Functional Magnetic Resonance Imaging
FPN: Frontoparietal Network
MDD: Major Depressive Disorder
rsFC: Resting-State Functional Connectivity
(r) TMS: (Repetitive) Transcranial Magnetic Stimulation
SNRI: Selective Serotonin and Norepinephrine Reupdate Inhibitor
SSRI: Selective Serotonin Reupdate Inhibitor
tDCS: Transcranial Direct Current Stimulation
VAN: Ventral Attention Network
