# Supplementary material for: Predicting the treatment outcomes of major depressive disorder interventions with baseline resting-state functional connectivity: a meta-analysis
Source: BMC Psychiatry. 2025 Apr 7;25:340. doi: 10.1186/s12888-025-06728-0 (PMC11974056; doi:10.1186/s12888-025-06728-0)
Supplement: Supplementary file 1 — Supplementary Material 1. [file 12888_2025_6728_MOESM1_ESM.docx]

**Supplement 1**

We used an eight network parcellation for this review based on the Human Brainnetome Atlas (Fan et al., 2016). As described by Zhang et al. (2023), the Humamn Brainnetome Atlas adds one additional network (i.e., the subcortical network) on top of the Yeo 7 Atlas (Yeo et al., 2011). The eight networks are: subcortical network (SCN), visual network (VN), somatosensory-motor network (SMN); dorsal attention network (DAN), ventral attention/salience network (VAN), frontoparietal/central executive network (FPN), limbic network (LN) and default mode network (DMN).

According to the Human Brainnetome Atlas, areas that fall under SCN include amygdala, hippocampus, basal ganglia and thalamus. Areas that fall under the VN include the fusiform gyrus and occipital cortex. Precentral gyrus, postcentral gyrus, paracentral lobule, and superior temporal gyrus fall under the SMN. DAN contains areas such as inferior temporal gyri. Nodes that fall under VAN include insula and inferior frontal gyrus. LN contains areas such as orbital frontal gyrus and superior temporal gyri. Areas that fall under the FPN include inferior parietal lobule and the frontal gyri. DMN contains areas such as the cingulate cortex, and precuneus.

Some areas such as the inferior parietal lobule and inferior temporal gyri fall under multiple networks. For situations like this, we refer to previous literature of MDD and assign the area to the network where the area is most frequently associated with. For areas not specifically entailed in the Human Brainnetome Atlas (Fan et al., 2016), we also referred to prior literature for categorization. For areas not mentioned in prior MDD literature, we referred to Yeo 7 atlas for guidance in categorization (Yeo et al., 2011).

Inferior parietal lobule, which also contains the angular gyrus, was categorized under the DMN (Kaiser, Andrews-Hanna, Wager, & Pizzagalli, 2015). Though the middle temporal gyrus examined in Tozzi et al. (2020) was categorized under the cognitive control network (an equivalent to FPN), it was categorized under the DMN based on the Yeo 7 atlas (Yeo et al., 2011). Cerebellum was assigned to FPN according to the classification mentioned in Kaiser et al. (2015). Striatum was assigned to VAN based on prior literature of MDD (Peters, Dunlop, & Downar, 2016). Supramarginal gyrus was categorized to the VAN based the Yeo 7 atlas. However, for Martens et al. (2021), where right angular gyrus is mixed with supramarginal gyrus, we categorize the predictive effect to DMN. The summary of the nodes categorization is summarized in Table 1.

*Table S1*. Networks examined and the nodes that fall under each network

| Name of the network | Nodes under the network |
| --- | --- |
| DMN | sgACC, L-PFC, vmPFC (BA10), distal ACC, rostral ACC, posterior cingulate cortex/precuneus, angular gyrus, middle temporal gyrus, middle temporal cortex, inferior parietal lobule |
| FPN | R-DLPFC, L-DLPFC (BA9, BA46), L-PFC, cerebellum |
| DAN | Superior frontal gyrus |
| VAN | Striatum, (anterior) insular cortex, supramarginal gyrus, transverse temporal gyrus, inferior frontal gyrus |
| SCN | Thalamus, parahippocampus, amygdala |
| SMN | SMA, premotor area, somatosensory association cortex, sensorimotor auditory network, precentral gyrus, postcentral gyrus |
| Visual | MT+ |

*Note*: DMN = default mode network; FPN = frontoparietal (central executive) network; DAN = dorsal attention network; VAN = ventral attention (salience) network; SCN = subcortical network; SMN = somatosensory-motor network; (sg)ACC = (subgenual) anterior cingulate cortex; L-PFC = left-prefrontal cortex; R/L-DLPFC = right/left-dorsolateral prefrontal cortex; SMA = supplementary motor area; MT+ = motion area MT in the visual cortex.

**Reference**

Fan, L., Li, H., Zhuo, J., Zhang, Y., Wang, J., Chen, L., . . . others (2016). The human brainnetome

atlas: a new brain atlas based on connectional architecture. *Cerebral cortex, 26* (8), 3508–3526.

Kaiser, R. H., Andrews-Hanna, J. R., Wager, T. D., & Pizzagalli, D. A. (2015). Large-scale network

dysfunction in major depressive disorder: a meta-analysis of resting-state functional

connectivity. *JAMA psychiatry, 72* (6), 603–611.

Martens, M. A., Filippini, N., Harmer, C. J., & Godlewska, B. R. (2021). Resting state

functional connectivity patterns as biomarkers of treatment response to escitalopram

in patients with major depressive disorder. *Psychopharmacology*, 1-14.

Peters, S. K., Dunlop, K., & Downar, J. (2016). Cortico-striatal-thalamic loop circuits of the

salience network: a central pathway in psychiatric disease and treatment. *Frontiers in systems*

*neuroscience, 10* , 104.

Tozzi, L., Goldstein-Piekarski, A. N., Korgaonkar, M. S., & Williams, L. M. (2020). Connectivity of

the cognitive control network during response inhibition as a predictive and response

biomarker in major depression: evidence from a randomized clinical trial. *Biological*

*psychiatry, 87* (5), 462–472.

Yeo, B. T., Krienen, F. M., Sepulcre, J., Sabuncu, M. R., Lashkari, D., Hollinshead, M., . . . others

(2011). The organization of the human cerebral cortex estimated by intrinsic functional

connectivity. *Journal of neurophysiology.*

Zhang, S., Zhou, J., Cui, J., Zhang, Z., Liu, R., Feng, Y., ... & Wang, G. (2023). Effects of

12‐week escitalopram treatment on resting‐state functional connectivity of large‐scale

brain networks in major depressive disorder. *Human Brain Mapping*, *44*(6), 2572-2584.
