# Supplementary material for: Predicting the treatment outcomes of major depressive disorder interventions with baseline resting-state functional connectivity: a meta-analysis
Source: BMC Psychiatry. 2025 Apr 7;25:340. doi: 10.1186/s12888-025-06728-0 (PMC11974056; doi:10.1186/s12888-025-06728-0)
Supplement: Supplementary file 2 — Supplementary Material 2. [file 12888_2025_6728_MOESM2_ESM.docx]

**Table S2**

*Nodes and their corresponding predictability from all reported articles*

| **Author** | **Treatment** | **Outcome Measure** | **Nodes** | **Pearson R** | **Spearman Rho** | **Regression coefficient** | **AUC** | **Cohen's d equivalent** | **N** | **95% CI original** | **95% CI Cohen's d** | **Connection Type Based on Human Brainnetome atlas** |
| --- | --- | --- | --- | --- | --- | --- | --- | --- | --- | --- | --- | --- |
| Avissar et al. (2017) | TMS | %△ in HRSD-24 | L-DLPFC <-> striatum | 0.58 |  |  |  | 1.424 | 27 | [0.2565266; 0.7866336] | [0.531; 2.551] | FPN - VAN |
|  |  |  | L-SMA+R-SMA+R-premotor+R-DLPFC+L-DLPFC+L-vmPFC+L-dACC | 0.693 (equivalent) |  |  |  | 1.922 |  | [0.4248773,  0.8493404] | [0.939; 3.218] | SMN - FPN, DMN |
| Cash et al. (2019) | rTMS | %△ in MADRS | DMN (seed: PCC) | -0.28 |  |  |  | -0.583 | 43 | [-0.535, 0.022] | [-1.266, 0.044] | DMN |
|  |  |  | Affective network (seed: subgenual cingulate) | -0.23 |  |  |  | -0.473 |  | [-0.496, 0.076] | [-1.142, 0.152] | DMN |
|  |  |  | Somatomotor control network | -0.21 |  |  |  | -0.43 |  | [-0.480, 0.096] | [-1.094, 0.193] | SMN |
| Cui et al. (2021) | escitalopram | △ in HRSD-17 | MTL subsystem | -0.33 |  |  |  | -0.699 | 36 | [-0.594122844, -0.001642317] | [-1.477; -0.003] | DMN |
| Elbau et al. (2023) | rTMS or iTBS | % △ in IQDS-SR | sgACC <-> L-DLPFC | -0.16 |  |  |  | -0.324 | 295 | [-0.26927772, -0.04665459] | [-0.559; -0.093] | DMN - FPN |
| Fu et al. (2021) | rTMS | HRSD reductive rate | L-DLPFC <-> L-anterior insular cortex | 0.66 |  |  |  | 1.757 | 27 | [0.3737179, 0.8314729] | [0.807; 2.988] | FPN - VAN |
|  |  |  | L-DLPFC <-> R-anterior insular cortex | 0.65 |  |  |  | 1.711 |  | [0.3585515 0.8259875] | [0.769; 2.931] | FPN - VAN |

| **Author** | **Treatment** | **Outcome Measure** | **Nodes** | **Pearson R** | **Spearman Rho** | **Regression coefficient** | **AUC** | **Cohen's d equivalent** | **N** | **95% CI original** | **95% CI Cohen's d** | **Connection Type Based on Human Brainnetome atlas** |
| --- | --- | --- | --- | --- | --- | --- | --- | --- | --- | --- | --- | --- |
| Ge et al. (2020) | iTBS OR HF-left stimulation | remission vs. non-remission | sgACC <-> DLPFC | 0.671 (equivalent) |  |  | 0.9 [0.81; 0.99] | 1.812 | 50 | [0.81, 0.99]; r [0.527, 0.854] |  | DMN - FPN |
|  |  | △ in HRSD | sgACC <-> R-DLPFC | -0.62 |  |  |  | -1.58 |  | [-0.7661320, -0.4129106] | [-2.384; -0.907] | DMN - FPN |
|  |  |  | sgACC <-> R-DLPFC | -0.65 |  |  |  | -1.711 | 24 | [-0.8345667, -0.3342449] | [-2.544; -1.018] | DMN - FPN |
|  |  | remission vs. non-remission | rACC - IPL | 0.447 (equivalent) |  |  | 0.76 [0.61; 0.89] | 0.999 | 50 | [0.61, 0.89]; r [0.194, 0.655] |  | DMN |
|  |  | △ in HRSD | rACC - IPL  rACC <-> L-lateral parietal cortex | 0.49 |  |  |  | 1.124 | 50 | [0.2450787, 0.6761301] | [0.505; 1.835] | DMN |
|  |  |  |  | 0.65 |  |  |  | 1.711 | 24 | [0.3342449, 0.8345667] | [1.019; 2.543] | DMN |
|  |  |  | rACC <-> MTC (middle temporal cortex) | 0.75 |  |  |  | 2.268 | 24 | [ 0.4969561, 0.8854930] | [1.484; 3.241] | DMN |
| Harel et al. (2024) | sertraline or fluoxetine or escitalopram | △ in HRSD-17 | Left amygdala – right amygdala | -0.33 |  |  |  | -0.699 | 62 | [-0.53562048,  -0.08743881] | [-1.269; -0.176] | SCN |
| Hsu et al. (2021) | sertraline | △ in HRSD-17 | Anterior insula <-> R-thalamus | 0.136 (equivalent) | 0.13 |  |  | 0.262 | 22 | [-0.3085180, 0.5229462] | [-0.649; 1.227] | VAN - SCN |
|  |  |  | parahippomcapus <-> R-inferior frontal gyrus | 0.052 (equivalent) | 0.05 |  |  | 0.1 |  | [-0.3796108, 0.4618720] | [-0.821; 1.041] | VAN - SCN |
|  |  |  | thalamus <-> L-superior frontal gyrus | 0.497 (equivalent) | 0.48 |  |  | 1.145 |  | [0.09537674,  0.75946964] | [0.192; 2.335] | SCN - DAN |
| Martens et al. (2021) | escitalopram | % △ in HRSD-17 | R-FPN <-> L-postcentral gyrus, L-precentral gyrus, BL precuneus and L-somatosensory association cortex | -0.601 |  |  |  | -1.504 | 34 | [-0.7805322,  -0.3298779] | [-2.497; -0.699] | FPN - SMN, DMN |

| **Author** | **Treatment** | **Outcome Measure** | **Nodes** | **Pearson R** | **Spearman Rho** | **Regression coefficient** | **AUC** | **Cohen's d equivalent** | **N** | **95% CI original** | **95% CI Cohen's d** | **Zones in Yeo 7 + Human Brainnetome atlas** |
| --- | --- | --- | --- | --- | --- | --- | --- | --- | --- | --- | --- | --- |
| Martens et al. (2021) | escitalopram | % △ in HAMD-17 | L-FPN <-> L-postcentral/precentral gyri | -0.412 |  |  |  | -0.904 | 34 | [-0.65843037,  -0.08578643] | [-1.750; -0.172] | FPN - SMN |
|  |  |  | Sensorimotor auditory network <-> R-supramarginal gyrus, R-angular gyrus, R-precuneus, posterior cingulate cortex | -0.365 |  |  |  | -0.784 |  | [-0.62590940,  -0.03061286] | [-1.605; -0.061] | SMN - VAN, DMN |
|  |  |  | DMN <-> R-angular and supramargial gyri | 0.371 |  |  |  | 0.799 |  | [0.03754463, 0.63011216] | [0.075; 1.623] | DMN - DMN, VAN |
|  |  | % △ in BDI | R-FPN <-> L-postcentral gyrus, L-precentral gyrus, BL precuneus and L-somatosensory association cortex | -0.614 |  |  |  | -1.556 |  | [-0.7884557,  -0.3481146] | [-2.564; -0.743] | FPN - SMN, DMN |
|  |  |  | L-FPN <-> L-postcentral/precentral gyri | -0.376 |  |  |  | -0.812 |  | [-0.63360290,  -0.04334572] | [-1.638; -0.087] | FPN - SMN |
|  |  |  | Sensorimotor auditory network <-> R-supramarginal gyrus, R-angular gyrus, R-precuneus, posterior cingulate cortex | -0.321 |  |  |  | -0.678 |  | [-0.59461909, 0.01925595] | [-1.479; 0.039] | SMN - VAN, DMN |
|  |  |  | DMN <-> R-angular and supramargial gyri | 0.327 |  |  |  | 0.692 |  | [-0.01255391, 0.59893532] | [-0.025; 1.496] | DMN - DMN, VAN |

| **Author** | **Treatment** | **Outcome Measure** | **Nodes** | **Pearson R** | **Spearman Rho** | **Regression coefficient** | **AUC** | **Cohen's d equivalent** | **N** | **95% CI original** | **95% CI Cohen's d** | **Zones in Yeo 7 + Human Brainnetome atlas** |
| --- | --- | --- | --- | --- | --- | --- | --- | --- | --- | --- | --- | --- |
| Moreno-Ortega et al. (2019) | ECT | △ in HRSD-21 | anterior DMN - DLPFC | 0.685 |  |  |  | 1.88 | 18 | [0.321,  0.873] | [0.678; 3.580] | DMN - FPN |
|  |  |  | Anterior DMN | -0.699 |  |  |  | -1.955 |  | [-0.8790115,  -0.3445814] | [-3.687; -0.734] | DMN |
|  |  |  | DLPFC - MT+ in visual network | -0.661 |  |  |  | -1.762 |  | [-0.8618899,  -0.2807785] | [-3.399; -0.585] | FPN - Visual |
| Raij et al. (2023) | HF-rTMS | % △ in BDI | BL. DLPFC | 0.44 |  |  |  | 0.98 | 25 | [0.05431158, 0.71144141] | [0.109; 2.025] | FPN |
| Wang et al. (2024) | Escitalopram | % △ in HRSD-17 | sgACC - L-DLPFC | 0.321 |  |  |  | 0.678 | 87 | [0.1183547, 0.4979761] | [0.238; 1.148] | DMN-FPN |
|  |  |  | sgACC – R-DLPFC | 0.376 |  |  |  | 0.812 |  | [0.1795747, 0.5435936] | [0.365; 1.295] | DMN-FPN |
|  |  |  | sgACC – L-inferior parietal lobule | 0.453 |  |  |  | 1.016 |  | [0.2679174, 0.6058371] | [0.556; 1.523] | DMN |
|  |  |  | sgACC – R-inferior parietal lobule | 0.428 |  |  |  | 0.947 |  | [0.2388894, 0.5858312] | [0.492; 1.446] | DMN |
| Weigand et al. (2018) | HF-rTMS | % △ in BDI | L-DLPFC <-> subgenual cingulate | -0.51 |  |  |  | -1.186 | 25 | [-0.7533236,  -0.1438591] | [-2.291; -0.291] | FPN - DMN |
| Ye et al. (2022) | escitalopram OR venlafaxine | HRSD-17 reductive ratio | R-angular among the DMN network | 0.42 |  |  |  | 0.926 | 66 | [0.1981054, 0.6009445] | [0.404; 1.504] | DMN |
| Zhang et al. (2021) | escitalopram OR sertraline OR fluoxetine | HRSD reductive ratio | distalACC (overlapped with BA25 and the top of BA4 at superior and inferior part) <-> L-DLPFC | 0.332 (converted) |  | 0.26 |  | 0.703 | 59 | [0.083;  0.542] | [0.167; 1.29] | DMN - FPN |
| Zhang et al. (2023) | escitalopram | HRSD reductive ratio | SCN <-> VAN | -0.3582913 (equivalent) | -0.344 |  |  | 0.768 | 41 | [-0.59982405,  -0.05691445] | [-1.499; -0.114] | SCN - VAN |
